# Evaluation of homocysteine, folate, vitamin B12, and vitamin D levels in pregnant women with recurrent vaginitis

**DOI:** 10.1590/1806-9282.20241284

**Published:** 2025-03-17

**Authors:** Gürkan Çıkım, Kemal Hansu

**Affiliations:** 1 Kahramanmaras Sutcu Imam University, Faculty of Medicine, Department of Medical Biochemistry – Kahramanmaraş, Turkey.; 2 Kahramanmaras Sutcu Imam University, Faculty of Medicine, Department of Obstetrics and Gynecology – Kahramanmaraş, Turkey.

**Keywords:** Vaginitis, Homocysteine, Folate, Vitamin B12, Vitamin D

## Abstract

**OBJECTIVE:**

The aim of this study was to evaluate the levels of homocysteine, vitamin B12, folic acid, and vitamin D in pregnant women with recurrent vaginitis and determine whether these parameters contribute to the etiology of the disease.

**METHODS:**

The study included 30 pregnant women diagnosed with recurrent vaginitis in their first trimester (group I), who presented at least twice between 1.5.2019 and 1.5.2022 at the obstetrics and gynecology clinic, and 30 healthy pregnant women in their first trimester without any complaints (group II). The vagititis group was compared with the control group for serum levels of vitamin B12, homocysteine, folic acid, and vitamin D.

**RESULTS:**

A comparison of the results between the groups revealed the following: homocysteine levels (μmol/L) were 10.75 (6.38–24.90) in group I (vaginitis positive) and 9.32 (4.26–17.10) in group II (control); vitamin B12 levels (ng/L) were 149.00 (63.00–328.00) in group I and 261.00 (126.00–544.00) in group II; folate levels (μg/L) were 10.56 (3.93–23.33) in group I and 9.48 (3.53–24.10) in group II; vitamin D levels (ng/mL) were 36.54 (23.65–75.68) in group I and 52.45 (26.57–105.00) in group II. Statistically significant elevation in homocysteine levels was observed in the vaginitis group (group I) (p<0.05). Vitamin B12 and D levels were significantly lower in the vaginitis group (group I) (p<0.05).

**CONCLUSION:**

In pregnant women with recurrent vaginitis, the levels of homocysteine, vitamin B12, and vitamin D may play a role in the etiology of the condition, and vitamins B12 and D may be considered for use in treatment.

## INTRODUCTION

Vaginitis is an infection and inflammation of the vaginal submucosa, mucosa, or both. The prevalence of vaginitis ranges between 11 and 20%^1^. Vaginitis can be classified based on its etiology into bacterial vaginosis (BV), vulvovaginal candidiasis (VVC), aerobic vaginitis, trichomonas vaginitis (TV), cytolytic vaginosis, and desquamative vaginitis^2^. Among these, BV has the highest prevalence (51%), followed by VVC (6.5%) and TV (2.5%)^3^. Risk factors include age, menstruation, multiple sexual partners, abortion, and poor hygiene. A healthy vaginal microbiome is maintained by the creation of an acidic vaginal pH by *Lactobacilli* ^4^. The importance of the vaginal microbiome, which provides a balance of the vaginal flora, is well known. Over the last decade, it has been determined that the microbiome is a significant contributing factor to human health and has an important role in women's reproductive health^5^. When this balance is disrupted, it creates a suitable environment for other infectious agents, leading to vaginitis. Vaginitis can cause various health issues such as pelvic inflammatory disease, sexually transmitted infections, urogenital infections, and an increased risk of abnormal pregnancy^6^. Symptoms include vulvovaginal discharge, itching, burning, foul odor, systemic pain, and dyspareunia. Diagnosis involves history taking, physical examination, staining, microscopy, culture, and DNA hybridization tests of vaginal discharge^7,8^. However, due to the presence of multiple infections or noninfectious factors, diagnosis usually prioritizes physical examination and symptoms.

Pregnancy involves numerous physiological and hormonal changes. Vaginitis in pregnancy poses several adverse risks to maternal and fetal health, including premature birth, miscarriage, intrauterine fetal death, amniotic fluid infections, and cervical insufficiency^9^. Homocysteine is an oxidative molecule formed during the metabolism of the amino acid methionine. It is implicated in the development of various diseases, including cardiovascular, cerebrovascular, renal, and thyroid disorders. Homocysteine is also linked to certain pregnancy complications^10^. Homocysteine is metabolized to cysteine via transsulfuration by the enzymes cystathionine beta-synthase and cystathionase, or via remethylation via the enzyme methionine synthase, dependent on vitamin B12 and folic acid, to methionine^11^. These two pathways are closely related. Several studies have demonstrated that homocysteine can induce cellular and molecular oxidative damage^12,13^. Vitamin B12, a water-soluble vitamin, is crucial for nerve conduction, DNA synthesis, and hematological processes^14^. Deficiency can lead to hyperhomocysteinemia, megaloblastic anemia, and neurological and cognitive disorders^15^. Folate plays a role in one-carbon unit transfer, methylation, and DNA and thymidylate synthesis. Its deficiency is associated with neural tube defects, growth and developmental delays, and hyperhomocysteinemia^16^. Vitamin D, a steroid derived from cholesterol, is involved in immune system regulation, bone development, and calcium/phosphorus balance and has anti-inflammatory properties^17^. Deficiency can lead to rickets, osteomalacia, diabetes, and cancer or contribute to these conditions^18^.

Pregnant women with recurrent vaginitis exhibit elevated homocysteine levels and reduced levels of vitamins B12 and D compared with healthy pregnant women. These changes in homocysteine, vitamin B12, and vitamin D levels may contribute to the development and persistence of recurrent vaginitis during pregnancy, potentially through mechanisms involving oxidative stress, impaired immune response, and tissue damage.

This study aims to investigate the levels of homocysteine, folic acid, and vitamin B12. These molecules are involved in oxidative processes. Additionally, it will examine vitamin D, which plays a role in immune system development and has anti-inflammatory effects. The study focuses on vaginitis during pregnancy, which can lead to serious outcomes. These include preterm birth, miscarriage, and intrauterine fetal death.

## METHODS

This study was conducted in accordance with the ethical approval from the Adıyaman University Ethics Committee, dated 15.3.2022, under decision number 2022/3-22, and written informed consent was obtained from each participant. The retrospective study included 30 pregnant women with recurrent vaginitis in their first trimester (group I), who presented at least twice with symptoms such as discharge, burning, foul odor, and itching between 1.5.2019 and 1.5.2022 at the Department of Obstetrics and Gynecology, Adıyaman University Training and Research Hospital, and 30 healthy pregnant women in their first trimester, matched for age (group II). Pregnant women with chronic diseases such as gestational diabetes and diabetes mellitus, those who smoked, and those who used any medication were excluded from the study. The study compared serum levels of homocysteine, vitamin D, folic acid, and vitamin B12. Venous blood samples were collected from both the patient and control groups after an overnight fast. Samples were centrifuged at 4,000 rpm for 10 min to separate the serum, and those not analyzed immediately were stored at −80°C. The assays were performed using the Beckman Coulter DxI 800 (Beckman Coulter, Kraemer Blvd. Brea, CA 92821, USA) for vitamin B12, vitamin D, and folic acid levels, and the Siemens Immulite analyzer (Diagnostic Products Corporation, Los Angeles, CA, USA) for homocysteine levels, utilizing electrochemiluminescence immunoassays. Exclusion criteria included women not in the first trimester of pregnancy, smokers, those using any medications, or with chronic diseases. Power analysis was performed to determine sample size. Using data from a pilot study, the effect size was calculated as 0.9781979. With a margin of error of 0.05 and a confidence level of 0.95, the required sample size was determined to be 60 (30 cases and 30 controls).

Statistical analysis was performed using IBM SPSS Statistics 22.0 (SPSS, Inc. Chicago, IL, USA). Normality of data distribution was assessed using the Kolmogorov-Smirnov test. As a result of the Kolmogorov-Smirnov test, Mann-Whitney U tests were used to compare group parameters as a result of data distribution not conforming to normality in the groups. Results are presented as med (min–max). A p-value of <0.05 was considered statistically significant.

## RESULTS

When comparing the results between groups, the levels of homocysteine (μmol/L) were found to be 10.75 (6.38–24.90) in group I (vaginitis positive) and 9.32 (4.26–17.10) in group II (control). Vitamin B12 levels (ng/L) were 149.00 (63.00–328.00) in group I and 261.00 (126.00–544.00) in group II. Folate levels (μg/L) were 10.56 (3.93–23.33) in group I and 9.48 (3.53–24.10) in group II. Vitamin D levels (ng/mL) were 36.54 (23.65–75.68) in group I and 52.45 (26.57–105.00) in group II. Statistically significant elevation in homocysteine levels was observed in the vaginitis group (group I) (p<0.05). Vitamin B12 and vitamin D levels were significantly lower in the vaginitis group (group I) (p<0.05). No statistically significant difference was found in folate levels between the groups (p>0.05). No significant difference was observed in ages between the groups (p>0.05) (Table 1).

**Table 1:** Comparison of pregnant women with recurrent vaginitis and healthy pregnant women.

Parameters	Control group (n=30)	Vaginitis group (n=30)
Age	31.27±6.81	30.52±6.81
Homocysteine	9.35±3.52	12.49±5.32*
Vit B12	284.44±110.45	169.08±76.25**
Folic acid	10.42±5.84	11.21±5.26
VitD	53.30±19.54	40.54±12.26**

In the vaginitis group, statistically higher levels of homocysteine and lower levels of vitamins B12 and D were observed compared with the control group (p<0.05, p<0.001, and p<0.001, respectively). There was no statistically significant difference in age and folate levels between the two groups (p>0.05).

* p<0.05 compared to control group.

** p<0.001 compared to control group.

## DISCUSSION

In a healthy vaginal flora, despite the presence of numerous microbial agents, approximately 95% of the flora consists of *Lactobacillus* species. Certain *Lactobacillus* species produce lactic acid and hydrogen peroxide (H_2_O_2_), which acidify the vaginal environment (pH<4.5) and thereby inhibit the colonization of other viruses and bacteria^19,20^. When this balance is disrupted, various infectious agents can colonize and cause vaginitis. Noninfectious vaginitis also exists. Vaginitis in pregnant women occurs with a prevalence ranging from 4 to 28%, depending on the etiology^21,22^. Vaginitis has been shown to increase the risk of severe complications in pregnancy, such as premature rupture of membranes, preterm birth, miscarriage, chorioamnionitis, neonatal infections, fetal death, sepsis, and maternal mortality^9^. Homocysteine is considered a component of the oxidative system and increases levels of asymmetric dimethylarginine, which inhibits nitric oxide synthase, leading to endothelial dysfunction^23^. Homocysteine has been demonstrated to damage vascular structures and cause cellular and molecular oxidative damage^12^. Additionally, deficiencies in folic acid and vitamin B12, which are involved in homocysteine metabolism, lead to hyperhomocysteinemia. There is limited literature on homocysteine levels in vaginitis. One study found elevated homocysteine levels and decreased vitamin B12 and folic acid levels in bacterial vaginitis^24^. Our study found a significant increase in homocysteine levels in the vaginitis group. We hypothesize that hyperhomocysteinemia, as an oxidative molecule, disrupts the structure of vaginal tissues and contributes to inflammation through endothelial dysfunction and pro-inflammatory effects, thus facilitating the development of vaginitis. Vitamin B12 is an important coenzyme in the elimination of homocysteine and is involved in DNA synthesis. Deficiency in B12 leads to neurological and cognitive disorders, hyperhomocysteinemia, megaloblastic anemia, pre-eclampsia, gestational diabetes (small for gestational age), FGR, and preterm births^15,16^. Our study observed reduced levels of vitamin B12 in pregnant women, which we attribute to increased requirements during pregnancy. We believe that decreased vitamin B12 contributes to hyperhomocysteinemia, thereby increasing oxidative stress, which negatively impacts vaginal tissues and facilitates vaginitis development. Additionally, reduced vitamin B12 may impair nucleic acid synthesis, thereby hindering tissue renewal and repair, predisposing to vaginitis. Folate deficiency is known to cause hyperhomocysteinemia and neural tube defects in pregnant women^16^. Our study did not detect any significant changes in folate levels between the groups. Vitamin D is essential for the development of multiple systems and functions, including the musculoskeletal system, immune system, cognitive capacity, and cardiovascular health^25^. In addition, it has many functions in the immune system, including cell proliferation, cellular differentiation, and endocrine. In pregnancy, it provides immune tolerance, prevents expulsion of the fetus, and also shows antimicrobial activity. BV is common in vitamin D deficiency. During pregnancy, calcium absorption from the maternal intestine increases by 35% due to the effect of vitamin D. Babies born to mothers with low vitamin D levels are prone to rickets (rickets). Various studies have shown that deficiency leads to premature birth and low birth weight. If there is a deficiency, it has been observed that babies born have learning and memory problems in later periods. VitD is effective in regulating insulin secretion and reducing insulin resistance by stimulating pancreatic β cells. VitD may play a role in the inflammation process in metabolic syndrome and DM with its anti-inflammatory activity. There is a significant relationship between vitamin D deficiency (<50 nmol/l) and gestational diabetes. Postpartum depression is more common in pregnant women with deficiency. Vitamin D requirements increase during pregnancy. Various studies have shown that vitamin D levels are either reduced or unchanged in pregnant women with and without vaginitis^26^. Our study found significantly lower vitamin D levels in pregnant women with vaginitis. We hypothesize that decreased vitamin D levels affect the immune system, weakening the defense mechanism and thereby contributing to the development of vaginitis.

In conclusion, we believe that it is important to prevent vaginitis during pregnancy, which contributes to the development of complications such as miscarriage, premature rupture of membranes, premature birth, and intrauterine fetal death, and to monitor homocysteine, vitamin B12, and vitamin D levels and eliminate vitamin deficiencies, if any, for a healthy pregnancy.
